# Study on the Effect of Freeze–Thaw Action on the Electrical Conductivity and Sensing Properties of Graphene-Based Cement Composites

**DOI:** 10.3390/ma16020855

**Published:** 2023-01-16

**Authors:** Huihui Chen, Ning Xu, Peng Jiang, Linhua Jiang

**Affiliations:** 1 School of Civil Engineering, Yancheng Institute of Technology, Yancheng 224051, China; 2 Nanjing Hydraulic Research Institute, Nanjing 210024, China; 3 College of Mechanics and Materials, Hohai University, Nanjing 210098, China

**Keywords:** freeze–thaw, graphene, cement-based composites, electrical conductivity, sensing property

## Abstract

Graphene can effectively improve the mechanical and electrical properties of cement-based materials due to its excellent tensile strength, thermal conductivity and electrical conductivity. In this paper, the effects of freeze–thaw on the conductivity and sensing properties of graphene-based cement materials were investigated. After the preparation of graphene-based cement materials, they were subjected to different times of freeze–thaw action. The experiments were designed to analyze the influence of freeze–thaw on the electrical conductivity, humidity sensitivity, thermosensitivity and pressure sensitivity of graphene-based cement composites. The results show that the influence of freeze–thaw on the electrical conductivity of graphene is mainly manifested in the influence on the resistivity and the extension of the polarization time, and the influence on the percolation transition zone is small. After freeze–thaw, the polarization time of the specimen decreases with the increase of the relative water content. The temperature has a great influence on the polarization effect of graphene-based cement composites and the composites with graphene content of the zone B still show satisfactory pressure-sensitive property after freeze–thaw.

## 1. Introduction

As one of the most important building materials in civil engineering, cement-based materials are widely used in infrastructure because of their wide range of sources and low prices. With the rapid development of science and technology, engineering structures are becoming larger and more complex, such as large sea crossing bridges, super high-rise buildings, large water conservancy projects, etc. The design service life of these major infrastructures is required to be high (usually more than 100 years). During the service period, under the combined action of long-term environmental erosion, material aging, long-term load effect, fatigue effect and other factors, common concrete damage such as cracking, deterioration and surface peeling is inevitable, and further reduce the service life of concrete structures. If not dealt with in time, it may lead to sudden structural damage, serious loss of life and property. Therefore, it has become a consensus to continuously monitor the performance of these cement composites in terms of stress, strain and damage indication, and to find and deal with the damage in time and establish the corresponding response mechanism [1,2,3,4,5].

Cement-based conductive composite can be used as a sensor, which can be poured synchronously with concrete, has good compatibility with concrete structure and deteriorates synchronously [6,7,8]. It overcomes the shortcomings of other sensors such as high installation requirements, poor compatibility and poor performance-matching, and better reflects the degree and process of structural degradation [9,10,11]. When necessary, it can also be directly used as a structural component to monitor the performance of the entire component without dead angles. So, the cement-based conductive composite is provided with broad application prospects [12,13,14,15,16,17]. Graphene [18] has become a new favorite for the preparation of cement-based conductive composites due to its excellent tensile strength, thermal conductivity and electrical conductivity, which can endow cement-based materials with certain functional properties.

Much research has been done on the conductive and sensing properties of graphene-based cement materials [19,20,21,22,23,24].

Bai et al. [25] studied the influences of graphene content, water/cement ratio, curing age and water content on the electrical conductivity of system. The results showed that when 0–2.5% of graphene were added, there was a percolation phenomenon in the graphene-based cement composites, and the percolation transition zone was 0.8~1.2%. Sedaghat et al. [26] found that the conductivity of cement paste was about 10^−8^ S/m. However, the incorporation of 1% graphene increases the conductivity by three orders of magnitude. The increase in graphene content seemed to be accompanied by changes in electrical properties from insulation to semiconductor behavior. Du et al. [27] investigated the electrical resistance of cement mortar mixed with 0, 1.2, 2.4, 3.6 and 4.8% of graphene by volume of cement-based composites. They found that the percolation threshold of graphene cement mortar was between 2.4 and 3.6%. Vojtěch Uher et al. [28] studied behaviors of electrically conductive cement composites with different dosages of electrically conductive fillers caused by various external conditions, including moisture, temperature and load. Kim et al. [29,30] found that adding an appropriate amount of silica fume (usually 10% of the cement mass) can promote the dispersion of nano-conductive materials such as carbon nanotubes and graphene, improve the pore structure, and further enhance the conductivity of cement-based composites. Li et al. [31] believed that the dispersion of GO was greatly improved by adding silica fume and GO to cement-based composites.

Cui et al. [32] reported that graphene nano sheets could effectively improve the thermal conductivity of cement-based composites, and the specific heat of composites decreased with the increase of graphene content. Zhao [33] studied the effect of graphene content on thermosensitivity and humidity sensitivity of graphene-based cement composites by the four-probe method. The results showed that the electrical resistivity of the composites decreased logarithmically with the increase of temperature, where the critical content of graphene was 0.8%. Ye et al. [34] observed that with the increase of rGO content, the thermal conductivity and thermal diffusivity coefficient of the specimen gradually increased.

Saafi [35] indicated that the resistivity of cement-based composites decreased with the increase of graphene content. Under load, the resistivity of composites decreased with the increase of compressive stress and increased with the increase of tensile stress. Cracks were generated in the matrix, and the resistivity of the matrix showed a sudden increase trend, which intuitively proved the pressure-sensitive property of graphene-based cement materials [36]. Zhang et al. [37] investigated the effect of graphene nanoplatelets and styrene-acrylate emulsion on the pressure sensitivity of cement materials and observed that the addition of graphene nanoplatelets could make the cement-based materials exhibit obvious pressure sensitivity. In addition, the structure of graphene nanoplatelets (C/O atomic ratio) had a great influence on the properties of cement composites. Danoglidis [38] developed multifunctional self-sensing cement mortar reinforced by multi-walled carbon nanotubes (MWCNT) and showed that the composites with 0.1 wt% MWCNTs give the highest fractional change in resistivity (10.6%) under external cyclic compressive loading. Parvaneh et al. [39] found that with the increase of carbon nanotube content, the electrical resistance and compressive strength of carbon nanotube-reinforced concretes decreased and increased, respectively. Moreover, when the content was 1 wt%, the smart performance was the best and the compressive strength increased from 42.47 to 80.33 MPa.

Although the conductive and sensing properties of graphene-based cement composites have been systematically studied, they mainly focus on the properties of new composites themselves, without considering the influence of freeze–thaw action on their conductive and sensing properties. In the long-term service process of cement-based materials, they are inevitably affected by freeze–thaw damage, which will change their composition, structure and performance. It can be predicted that freeze–thaw action will have an important impact on the conductivity and sensing properties of graphene-based cement materials. In this paper, graphene-based cement composites were taken as the research object, and experiments were designed to investigate the effects of freeze–thaw on the electrical conductivity, humidity sensitivity, thermosensitivity and pressure sensitivity ofgraphene-based cement composites. The influence mechanism of freeze–thaw on the electrical conductivity of composites was analyzed by SEM test method. The influence of freeze–thaw action, relative water content and temperature on the resistivity of composites were also explored.

## 2. Materials and Methods

### 2.1. Materials

#### 2.1.1. Cement

The cement was P. II 42.5 Portland cement. All performance indexes of cement meet the requirements of Chinese Standard GB175-2007, and its chemical composition and physical properties are shown in Table 1 and Table 2, respectively.

#### 2.1.2. Silica Fume

The silica fume used in this study had a SiO_2_ content of more than 85%, an average particle size of about 0.2 μm, and a specific surface area of 26.4 m^2^/g. Its chemical composition is listed in Table 3.

#### 2.1.3. Graphene

**Figure 1:**
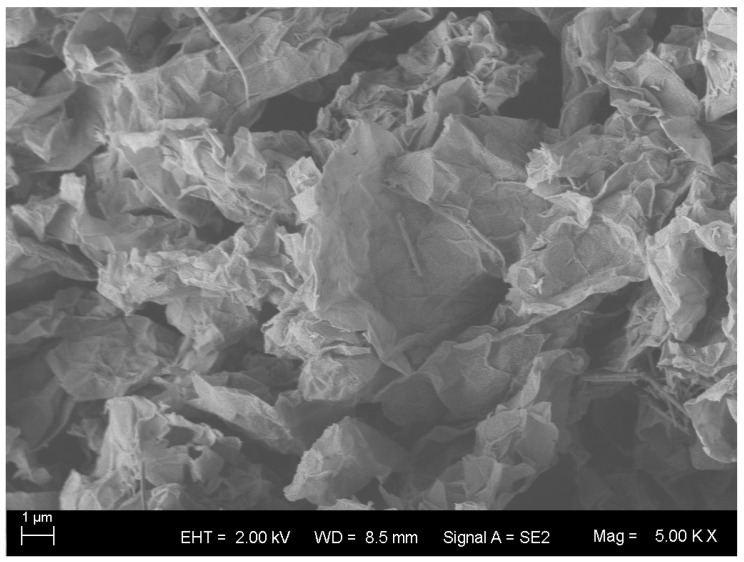
Microtopography of graphene.

The graphene powder was purchased from Shenzhen Guosen Linghang Technology Co., Ltd. (China), and prepared by physical exfoliation method. The micromorphology is presented in Figure 1, and the relevant performance indexes are listed in Table 4.

#### 2.1.4. Mixing Water

The mixing water was the tap water of the laboratory, which met the requirements of the Chinese standard of water for concrete.

#### 2.1.5. Admixtures

Admixtures included graphene dispersant (sodium dodecyl benzene sulfonate), superplasticizer (polycarboxylic acid type) and defoamer (silicone type).

### 2.2. Specimen Preparation

The mix proportions of graphene-based cement composites are shown in Table 5. In order to better disperse graphene and improve the conductivity of cement-based composites [29], the ratio of silica fume to cement was selected as 1:9. In addition, the use of silica fume helps to improve the durability of graphene-based cement composites.

Graphene dispersion was pre-prepared according to the mix proportions in Table 5. The required mass of dispersant, superplasticizer, defoamer and water were poured into a beaker, mixed, stirred with a glass rod for two minutes, and left to stand for use. The required mass of graphene powder was added to the mixed solution and magnetically stirred by magnetic stirrer for 10 min, so that the graphene powder was completely immersed in the mixed solution. Then the beaker was put into an ultrasonic cell disruptor, and ultrasonically treated at room temperature for 50 min to obtain the graphene dispersion.

According to the mix proportions in Table 5, the required mass of cement and silica fume was poured into the paste mixer and mixed evenly, and the pre-prepared graphene dispersion was poured while mixing. After sufficient mixing, the paste was poured into the mold and shaken properly to eliminate the bubbles mixed in the paste. The size of the specimen was 20 × 20 × 60 mm. The specimens were demoulded 24 h after molding and cured in water for three months to ensure full hydration of the cement and silica fume.

### 2.3. Test Methods

#### 2.3.1. Freeze–Thaw Test

After the specimens were cured, they were put into a water tank, and tap water was injected to cover the specimens by more than 20 mm. The water tank was then put into a low-temperature freezer for freezing, and the temperature was set to −20 °C. When the water tank was fully frozen, it was taken out and thawed at room temperature. Thus, a freeze–thaw cycle was completed. The specimens were taken out for other tests while 25, 50, 75 and 100 cycles were accomplished.

#### 2.3.2. Electrical Resistivity Measurement

Since freeze–thaw is easy to cause the specimen to break along the stainless-steel gauze used in direct current (DC) four-probe method, DC two-probe method was employed to measure the electrical resistivity of specimen after freeze–thaw in this study [30,40].

After curing and freeze–thaw tests, conductive copper foil paper was pasted on the two ends of the specimen, and the wire was led to measure the voltage and current at two ends of the specimen. The electrical resistivity *ρ* can be obtained as follows:

(1) $$\rho =R\cdot \frac{S_{0}}{L}=\frac{U}{I}\cdot \frac{S_{0}}{L},$$

where *R*, *U* and *I* are the resistance, voltage and current at both ends of the specimen, respectively, *S*_0_ represents the sectional area of the specimen and *L* is the length between the two ends.

#### 2.3.3. Humidity Sensitivity Test

The humidity sensitivity of the specimen is the relationship between the resistivity and the internal moisture content of the specimen. The relative water content of the specimen is calculated as follows:

(2) $$S=\frac{m_{S}-{m_{0}}^{\prime }}{m_{0}-{m_{0}}^{\prime }},$$

where *S* is the relative water content of the specimen, *m*_s_ is the mass of the specimen when its relative water content is *S*, *m*_0_ and *m*_0_′ are the mass when the specimen is saturated and dried, respectively.

In order to prepare the specimen with the desired relative water content, the mass of the specimen was measured first when it was dried and saturated, respectively. Then the specimen was wrapped with plastic wrap to prevent the increase or loss of water content while the desired relative water content was reached and left to stand for one month to make the moisture inside the specimen relatively uniform and stable.

#### 2.3.4. Thermosensitivity Test

The thermosensitivity of the specimen is the relationship between the resistivity and the temperature of the specimen. The specimen was dried and cooled to room temperature and wrapped with plastic wrap to prevent the specimen from absorbing water. If the ambient temperature was above zero, a water bath was used to set the test temperature. A low temperature freezer was adopted to set the test temperature if the ambient temperature was below zero. The specimen was placed in the environment at the set temperature for more than five hours to achieve the same internal and external temperature. The resistivity of the specimen was measured after reaching the set temperature.

#### 2.3.5. Pressure Sensitivity Test

The specimen with the desired temperature and humidity was placed vertically on the universal testing machine, and the place in contact with the indenter was separated by an insulating gasket to prevent leakage. Then the resistivity measurement equipment was connected, and the ammeter and voltmeter (Fluke multimeter) were connected to the computer to record the whole process of current and voltage changes under compression. Before the universal testing machine started, the resistivity of the specimen was measured first to complete the polarization. When the resistivity was stabilized, the universal testing machine was set to load at a rate of 0.2 kN/s to crush the specimen and the pressure-time curve was recorded in real time. If the resistivity under cyclic loading was measured, different maximum loads and loading rates were set: For the load rate was 0.2 kN/s, the maximum loads were 1, 2 and 3 kN, respectively, and for the maximum load was 2 kN, the corresponding loading rates were 0.2, 0.5 and 1 kN/s, respectively.

## 3. Results and Discussion

### 3.1. Electrical Conductivity

#### 3.1.1. Polarization Effect

**Figure 2:**
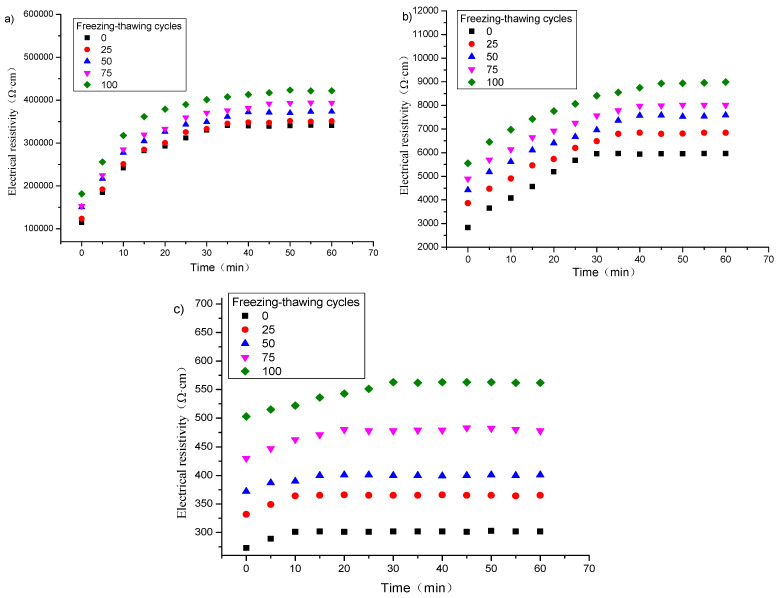
Polarization effect of specimens with different graphene content after different freezing and thawing cycles: (**a**) 1%; (**b**) 2%; (**c**) 3%.

Figure 2 shows the change of electrical resistivity with time of specimens with graphene content of 1, 2 and 3% after different freeze–thaw cycles. It can be seen from Figure 2, that for the specimens with the same graphene content, the number of freeze–thaw cycles has little effect on the shape of the resistivity-time curve. Furthermore, the initial resistivity and the stable resistivity gradually increase with the increase of the number of freeze–thaw cycles.

The polarization time obtained from Figure 2 is listed in Table 6. It can be observed that for specimens containing the same graphene content, the polarization time of each specimen increases with the increase of freeze–thaw cycles.

#### 3.1.2. Percolation Threshold

**Figure 3:**
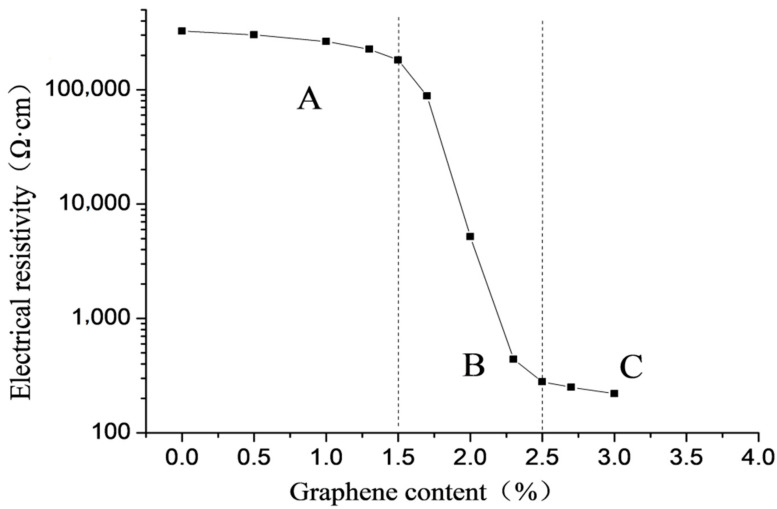
Relationship between electrical resistivity and graphene content of specimens without freeze–thaw action.

The change of the electrical resistivity of the specimen not subjected to freeze–thaw action with the content of graphene is plotted in Figure 3. The resistivity in this case was measured by DC four-probe method [27]. It is noted that the graphene content can be roughly divided into three zones according to variation curve of electrical resistivity with graphene content. Zone A refers to the range of 0 to 1.5% of the content of graphene, which is characterized by small influence of the content of graphene on the electrical resistivity, and large electrical resistivity of the specimen. The range of graphene content between 1.5 and 2.5% is defined as the percolation transition zone, and expressed as zone B. The characteristic of this zone is that the resistivity decreases sharply with the increase of graphene content, which can eventually be reduced by three orders of magnitude. Zone C indicates the range from more than 2.5% to a certain amount of graphene content. The feature of this zone is that the graphene content has little effect on the resistivity and the resistivity of the specimen is small. Graphene content of 1.5 and 2.5% are the upper and lower thresholds of the percolation transition zone, respectively.

**Figure 4:**
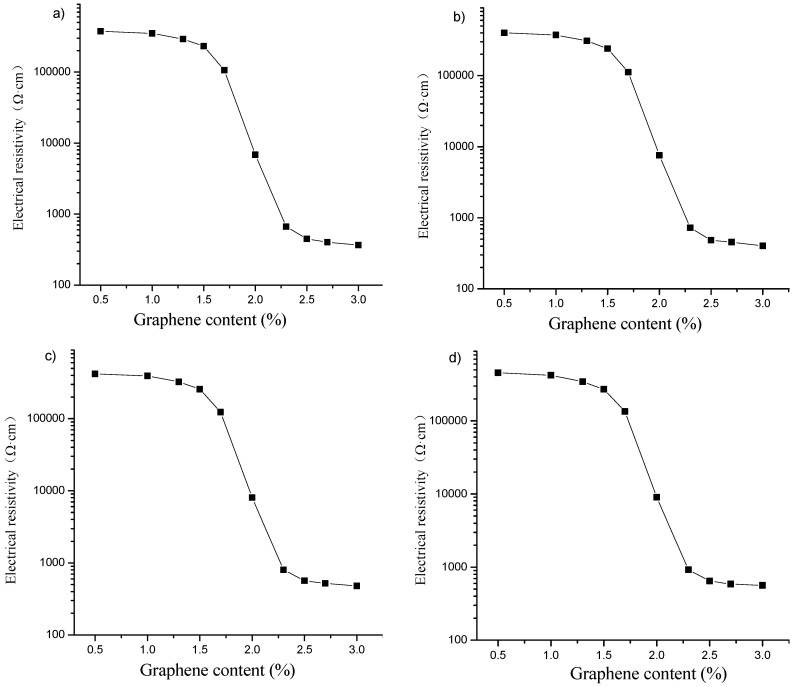
Electrical resistivities of specimens with different graphene contents after different freeze–thaw cycles: (**a**) 25; (**b**) 50; (**c**) 75; (**d**) 100.

Figure 4 represents electrical resistivities of specimens with different graphene contents after different freeze–thaw cycles. The results are similar to those of the specimens not subjected to freeze–thaw action in Figure 3. During the freeze–thaw cycle, the porosity in the cement matrix increases. In the case of the same graphene content, the electrical resistivities of the specimens increase with the number of freeze–thaw cycles, still on an order of magnitude.

**Figure 5:**
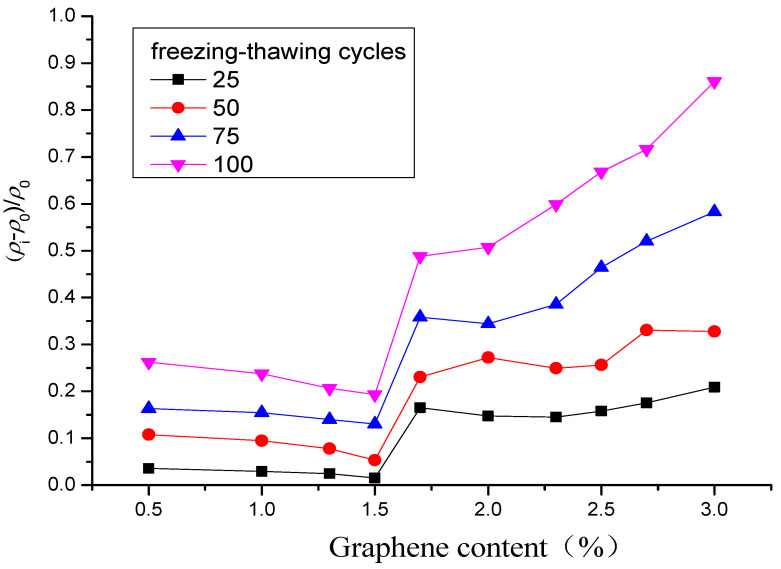
Relative change rate of electrical resistivity after different freeze–thaw cycles.

Figure 5 shows the relative change rate of electrical resistivity of the specimen after different freeze–thaw cycles. As shown in Figure 5, in the case of the same graphene content, the relative change rate of electrical resistivity of the specimen increases gradually with the increase of the number of freeze–thaw cycles, and they are all greater than zero. When the graphene content is in zone A, when the content of graphene is in zone A, graphene is isolated and dispersed in the specimen, and the gap between particles is large. The specimen mainly depends on the ion conduction in the cement matrix. At this time, the freeze–thaw causes the matrix to produce new cracks, and the pore size of the cracks increases, reducing the ionic conductivity and increasing the resistivity. Under the same number of freeze–thaw cycles, with the increase of graphene content, the freeze–thaw resistance of the specimen enhances, and the relative change rate of resistivity decreases. When the graphene content is in zone B, the distance between graphene particles decreases gradually with the increase of graphene content. When the particles are close enough, although there is no contact, the carriers can obtain enough energy from the outside to arrive at another conductor from one conductor, and electronic transition occurs to form tunneling conduction, which is dominant in zone B. The cracks caused by freeze–thaw increase the distance of some graphene particles, destroy their occurrence of tunneling conduction, and increase the resistivity. This is essentially different from the influence of freeze–thaw on the resistivity of zone A, and the influence is greater. Thus, under the same number of freeze–thaw cycles, the relative change rate of resistivity first increases rapidly and then stabilizes with the increase of the graphene content. When the graphene content is in zone C, the graphene particles have lapped with each other to form a better conductive network. Contacting conduction is dominant for the conduction of the composite. Like zone B, cracks caused by freeze–thaw destroy the contact of graphene particles, which in turn leads to changes in the conductive network and resistivity. In addition, the relative change rate of resistivity increases faster with the increase of the number of freeze–thaw cycles, and the damage effect of freeze–thaw on the complete graphene conductive network is more obvious [25,41].

#### 3.1.3. Microscopic Analysis of the Effect of Freeze–Thaw on Electrical Conductivity

**Figure 6:**
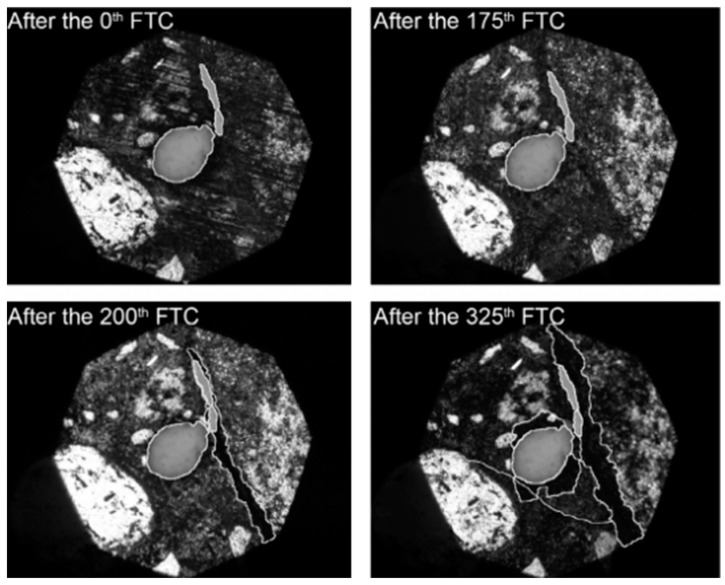
Evolution of pore structure in cement matrix caused by freezing and thawing [42].

The freeze–thaw damage goes from the surface to the inside. Literature [42] had investigated the evolution process of the pore structure of matrix under freeze–thaw action, as illustrated in Figure 6. As the number of freeze–thaw cycles increases, the cement matrix is damaged more and more seriously. Consequently, the pore size increases in the pore structure, the connected pores become thicker, the number increases, and the small pores are connected to each other.

**Figure 7:**
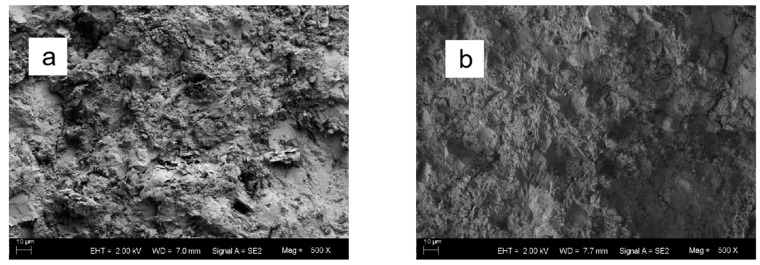

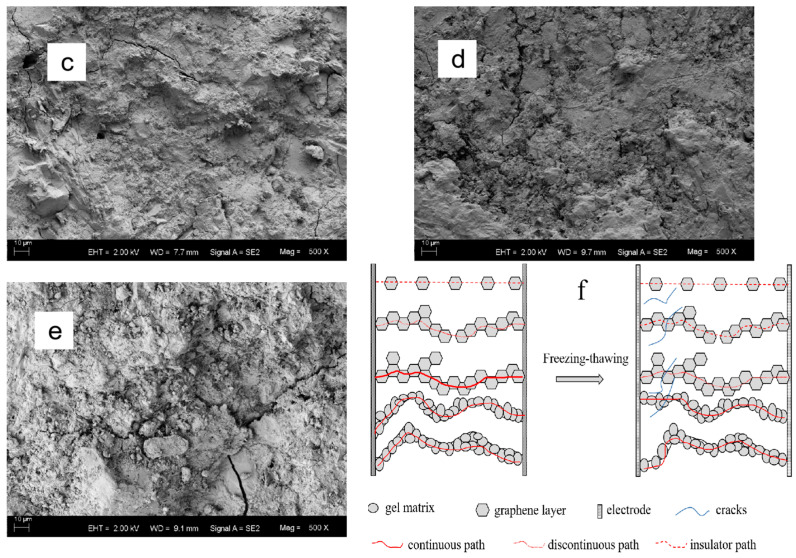
SEM images of graphene-based cement composites and schematic plot of conductive path after different freezing and thawing times: (**a**) 0; (**b**) 25; (**c**) 50; (**d**) 75; (**e**) 100; (**f**) schematic plot.

Figure 7 depicts the SEM images and conductive path schematic plot of the graphene-based cement composites after freeze–thaw cycles. As observed, the left figure in Figure 7f illustrates several conductive paths in the composite before freeze–thaw. The first path above is the insulation path. At this time, the graphene particles are independent and sparsely distributed. The second is a discontinuous conductive path, in which the graphene clusters close to each other or in contact form a tunneling conduction. The third is the contacting conduction path. The graphene particles contact each other and stack continuously to form a chain, generating a continuous conductive path. The bottom two paths are ionic conduction of the gel matrix with low conductivity. After freezing and thawing, as shown in the right figure of Figure 7f, the water in the pores freezes and expands, making the pore size larger, generating new cracks, and increasing the porosity of the matrix. With the increase of freeze–thaw cycles, the matrix cracks increase, the crack width becomes larger, and the gel becomes loose. The crack increases the distance between the adjacent graphene clusters, which makes it impossible to form tunneling conduction. Additionally, the crack also destroys the continuity of the graphene in contact with each other in the matrix. As a consequence, the resistivity of the composite increases.

### 3.2. Humidity Sensitivity Property

#### 3.2.1. Influence of Freeze–Thaw on Polarization Effect under Different Humidity

**Figure 8:**
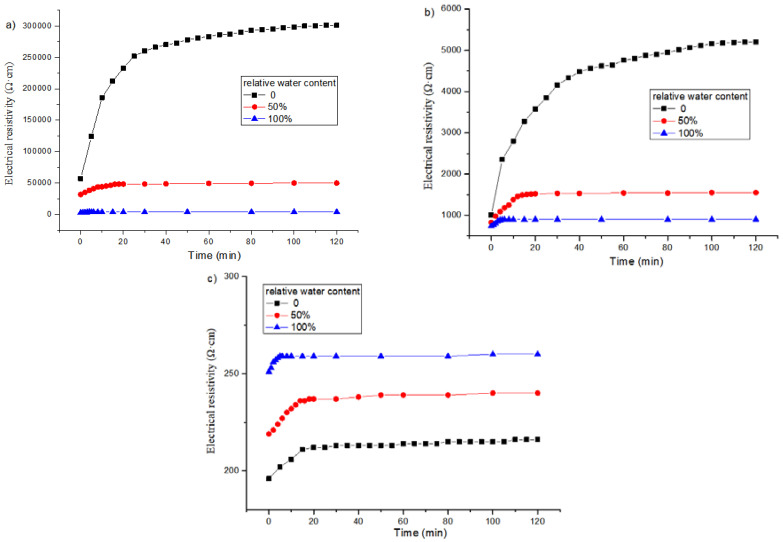
Influence of relative water content on polarization effect of specimens with different graphene content without freeze–thaw cycles: (**a**) 1%; (**b**) 2%; (**c**) 3%.

Figure 8 shows the electrical resistivity changes with time under different relative water contents for the specimens with 1, 2 and 3% content of graphene without freeze–thaw cycles. It can be found that when the graphene content is 1 or 2%, the polarization curve of the specimen in the wet state becomes gentle, the difference between the initial resistivity and the stable resistivity becomes smaller, and the final stable value decreases. Moreover, the greater the relative water content, the more obvious the effect. When the content of graphene is 3%, the effect of relative water content on the polarization effect is opposite.

**Figure 9:**
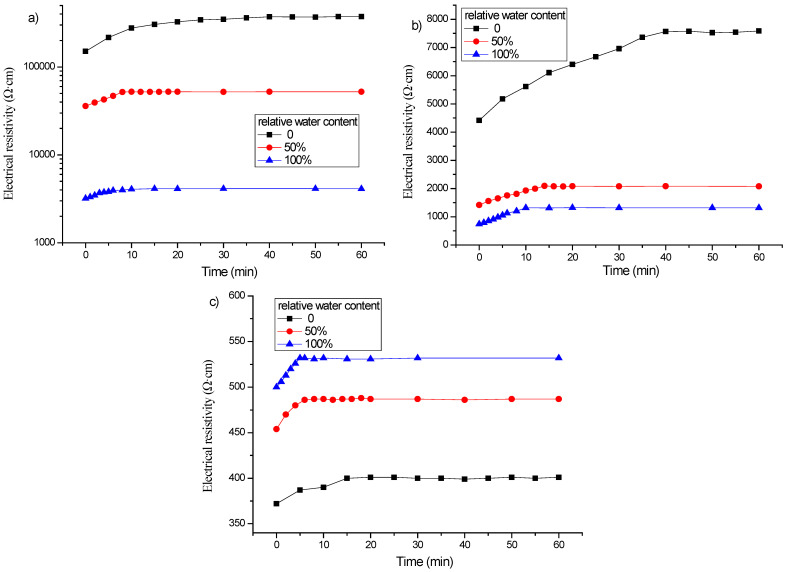
Influence of relative water content on polarization effect of specimens incorporating different graphene contents after 50 freeze–thaw cycles: (**a**) 1%; (**b**) 2%; (**c**) 3%.

Figure 9 is a graph showing the change of electrical resistivity with time of specimens with graphene contents of 1, 2 and 3% and different relative water contents after 50 freeze–thaw cycles, and the corresponding polarization time is listed in Table 7. It appears that at 50 or 100% relative water content, the polarization effect of the electrical resistivity of the specimen after freeze–thaw is similar to that without freeze–thaw. When the graphene content is 1%, after 50 freeze–thaw cycles, the porosity increases due to freeze–thaw, the conductive network of the pore solution is enhanced, and the resistance of the pore solution decreases. When the content of graphene is 2%, the polarization effect is also jointly controlled by the graphene tunnel and the conductive network of pore solution during the freeze–thaw process. While the graphene content is 3%, the conductivity of the specimen mainly depends on the graphene conductive network, and the polarization effect is not greatly affected by the relative water content and freeze–thaw.

#### 3.2.2. Effect of Freeze–Thaw on Electrical Resistivity under Different Humidity

**Figure 10:**
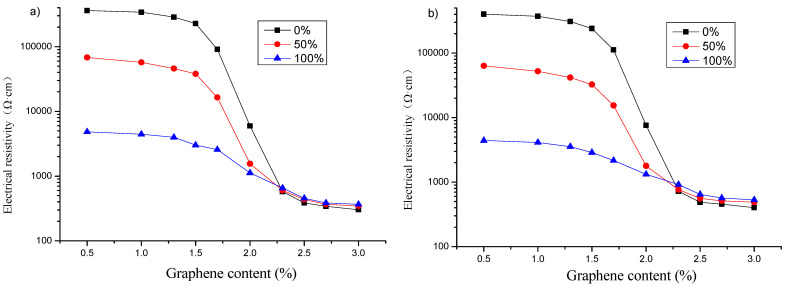
Effect of relative water content on electrical resistivity of specimens with different graphene content: (**a**) before 50 freeze–thaw cycles; (**b**) after 50 freeze–thaw cycles.

**Figure 11:**
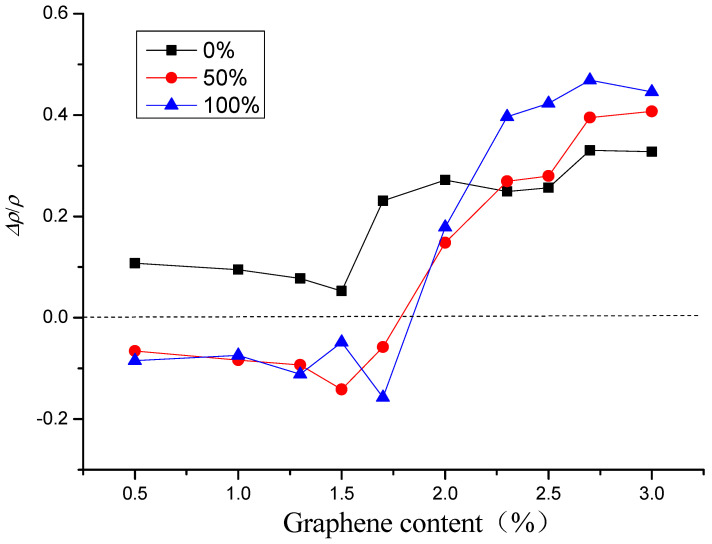
Relative change rates of electrical resistivity of specimens with different graphene content after freeze–thaw.

The effect of relative water content on the electrical resistivity of graphene-based cement composites before and after 50 freeze–thaw cycles is displayed in Figure 10. The relative change rates of electrical resistivity of specimens after freeze–thaw with the same relative water content are presented in Figure 11. Comparing the results before and after freeze–thaw, it can be observed that for the specimen with relative water content of 50% or 100%, its electrical resistivity after 50 freeze–thaw cycles decreases when the graphene content is in zone A, and changes from decreasing to increasing in zone B. The electrical resistivity of the specimen increases when the graphene content is in zone C, and increases with the increase of graphene content. It can also be concluded from Figure 10 that the freeze–thaw action does not change the influence trend of relative water content on resistivity, and the improved Logistic function [25] can still be used to express the relationship between electrical resistivity and relative water content after freeze–thaw action.

As presented in Figure 11, when the content of graphene is in the range of 0~1.7%, the relative change rate of electrical resistivity after freeze–thaw in dry state is greater than zero, for the relative water content of 50 or 100%, the relative change rates of resistivity in both cases are close to and both less than zero. When the graphene content is greater than 1.7%, all the relative change rates of resistivity increase rapidly. As the graphene content continues to increase, finally, the greater the relative water content, the greater the relative change rate of resistivity.

For the specimens with graphene content of zone A and relative water content of 50 and 100%, respectively, it is mainly ionic conduction, and the conductive path is composed of a large amount of pore solution in the connected pores. The microcracks caused by freeze–thaw increase the porosity, enhance the conductive network of pore solution, and reduce the resistance of pore solution, resulting in a negative relative change rate of resistivity. At the same time, due to the freezing and melting of water in the pores, some ions are free to the outside of the matrix, and the content of conductive ions in the pores decreases. Under the combined effect of the two, the relative change rates of the resistivity of the specimens are similar when the relative water content is 50 and 100%, respectively. In the case of the specimens containing graphene content of zone B, the ionic conduction of the pore solution and tunneling conduction play a role together. With the increase of graphene content, tunneling conduction gradually plays a leading role. Freeze–thaw produces more pores or microcracks, which increases the spacing of graphene particles, resulting in a decrease in the conductivity of tunneling conduction. The relative change rates of resistivity of specimens increase rapidly with the increase of graphene content. For the specimens incorporating graphene content of zone C, contacting conduction is the main conductive form. The concentration of graphene particles is greater than the percolation threshold. And the particles overlap each other to form a complete conductive network, by which the conductivity is carried out. The cracks caused by freeze–thaw destroy the contact of graphene particles, which can reduce the conductivity of graphene network. Especially when the number of freeze–thaw cycle is more, the specimen is seriously damaged, and the electrical resistivity of the specimen is increased [43,44].

**Figure 12:**
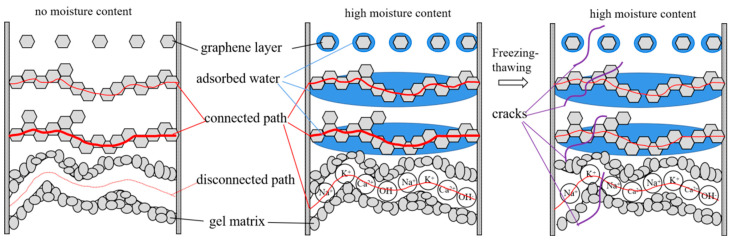
Microstructure schematic diagram of effect of humidity on electrical conductivity of graphene-based cement composites after freeze–thaw.

Figure 12 displays the microstructure analysis diagram of the effect of humidity on the electrical conductivity of graphene-based cement composites under freeze–thaw action. It can be seen from the left figure of Figure 12, that in the dry state, there are cement gel matrixes, close graphene and complete graphene chains in the composite that can conduct electricity. When a large amount of water is contained, as shown in the middle figure of Figure 12, pore solution in the matrix dissolves free ions such as Na^+^, K^+^, Ca^2+^ and OH^−^ to form ion conduction, which can reduce the resistivity of the matrix by two orders of magnitude. At the same time, due to the huge specific surface area of graphene, water will be adsorbed on the graphene surface to form a layer of water film. When the water film is on graphene which is close to each other, it can reduce the resistance of electron transition and enhance the tunneling conduction effect. In the complete graphene contact conductive network, however, the water film will increase the resistance of free electrons to directional movement under the action of electric field, resulting in an increase in resistivity. After freezing and thawing, as shown in the right figure of Figure 12, the generation of new cracks and the increase of porosity enhance the ionic conductivity of pore solution. On the other hand, the distance between graphene is increased, the continuity of graphene in contact is destroyed, weakening the tunneling conduction and contacting conduction.

### 3.3. Thermosensitive Property

#### 3.3.1. Influence of Freeze–Thaw on Polarization Effect at Different Temperatures

**Figure 13:**
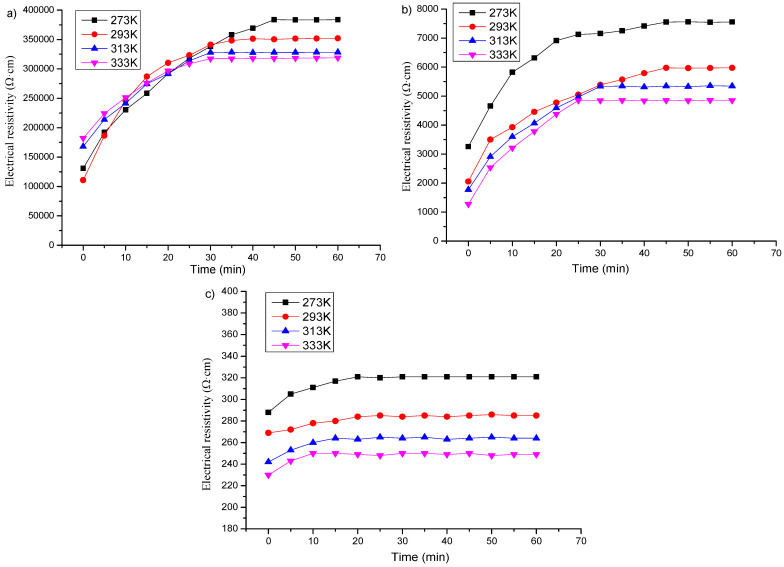
Influence of temperature on polarization effect of specimens with different graphene contents after freeze–thaw: (**a**) 1%; (**b**) 2%; (**c**) 3%.

Figure 13 shows the change of electrical resistivity of specimens with graphene content of 1% (in zone A), 2% (in zone B) and 3% (in zone C) at different temperatures after 50 freeze–thaw cycles, and the final polarization time is listed in Table 8. It can be concluded from Figure 13 and Table 8, that after freeze–thaw, the higher the temperature of each specimen, the faster the polarization, and the smaller the resistivity value after stabilization.

#### 3.3.2. Influence of Freeze–Thaw on Electrical Resistivity under a Single Heating

**Figure 14:**
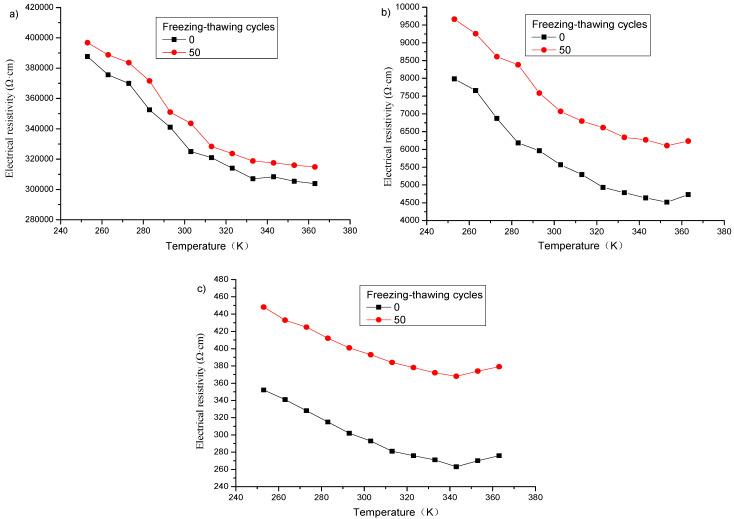
Influence of temperature on electrical resistivity of specimens with different graphene contents before and after freezing and thawing: (**a**) 1%; (**b**) 2%; (**c**) 3%.

Figure 14 depicts the electrical resistivity changes of specimens with graphene content of 1% (in zone A), 2% (in zone B) and 3% (in zone C) in a single heating between 253 and 363 K after different freeze–thaw cycles. As indicated in Figure 14, the electrical resistivity of the specimen containing 1% of graphene shows a negative temperature coefficient property with the increase of temperature in the whole test temperature range. The electrical resistivity of the specimen after freeze–thaw is greater than that of the specimen without freeze–thaw at each measuring temperature. When the temperature is lower than 353 K, the resistivity of the specimen incorporating 2% of graphene after freeze–thaw represents a negative temperature coefficient property with the change of temperature. However, when the temperature exceeds 353 K, the specimen incorporating 2% of graphene exhibits a positive temperature coefficient property. This phenomenon is more obvious in the specimen with 3% content of graphene. The positive temperature coefficient property begins to appear when the temperature just reaches 343 K.

For graphene-based cement composites, there are two main reasons for the influence of temperature on its resistivity. On the one hand, the increase of temperature can increase the concentration and migration rate of carriers in the material and decrease the resistivity. On the other hand, the material volume changes due to temperature rise. The thermal expansion coefficient of cement matrix is about 15 × 10^−6^/K, while the thermal expansion coefficient of graphene is about −4 × 10^−6^/K [45] within the test temperature range. This volume change difference increases the distance between graphene particles, leading to an increase in resistivity. These two temperature influence mechanisms work at the same time. When the content of graphene is 1%, the distance between graphene particles is far. The volume change caused by temperature rise has little effect on the resistivity of the specimen, and the thermal motion of carriers is intensified, thus reducing the resistivity of the specimen. When the graphene content is 2%, the distance between the graphene particles is close. The tunneling conduction can be carried out, and the conduction effect is enhanced after the temperature rises. Meanwhile, the volume of graphene particles becomes smaller, and the cement matrix expands, resulting in an increase in the distance between graphene particles and a decrease in tunneling conductivity. Under the combined action of these two mechanisms, the thermal motion enhancing conductivity dominates between 273 and 353 K, and the volume change dominates when the temperature exceeds 353 K. Similarly, when the graphene content is 3%, a complete graphene conductive network is formed, and the temperature at which the influence mechanism of temperature rise on the resistivity of the specimen changes from thermal motion to volume change is reduced to 343 K. After freezing and thawing, the porosity of the specimen is increased, and the cracks generated destroy the conductive path, making the resistivity increase. However, the influence trend of temperature on the resistivity of graphene-based cement composites is not destroyed [28,44].

According to literatures [46,47] and the results of Figure 14, the relationship between electrical resistivity and temperature of graphene-based cement composites before and after freeze–thaw can be fitted with the following expression:

(3) $$\rho =\rho_{m}e^{\frac{b}{T}},$$

where *ρ* is the electrical resistivity, *ρ_m_* and *b* are the fitting coefficients, and *T* is the absolute temperature.

**Figure 15:**
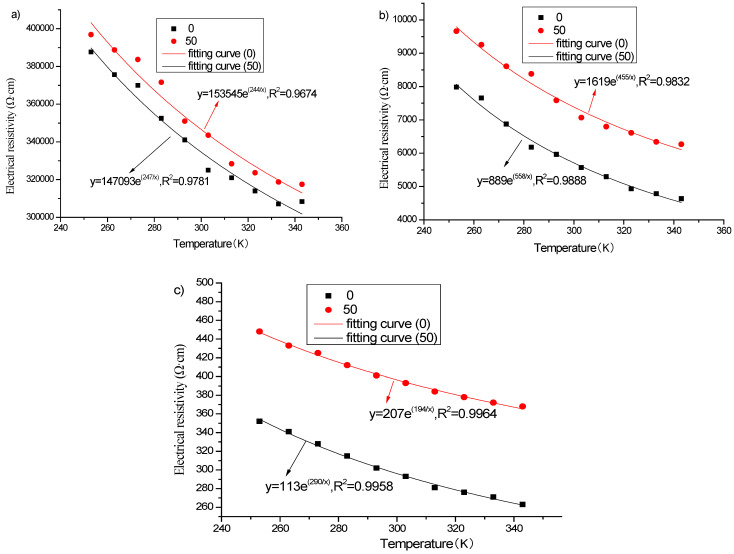
Fitting of electrical resistivity of specimens with different graphene contents before and after freeze–thaw: (**a**) 1%; (**b**) 2%; (**c**) 3%.

The fitting results are shown in Figure 15 and Table 9 and indicate that there is still a satisfactory exponential function relationship between the electrical resistivity and temperature of graphene-based cement composites after freeze–thaw.

#### 3.3.3. Influence of Freeze–Thaw on Electrical Resistivity under Cyclic Temperature Rise and Fall

**Figure 16:**
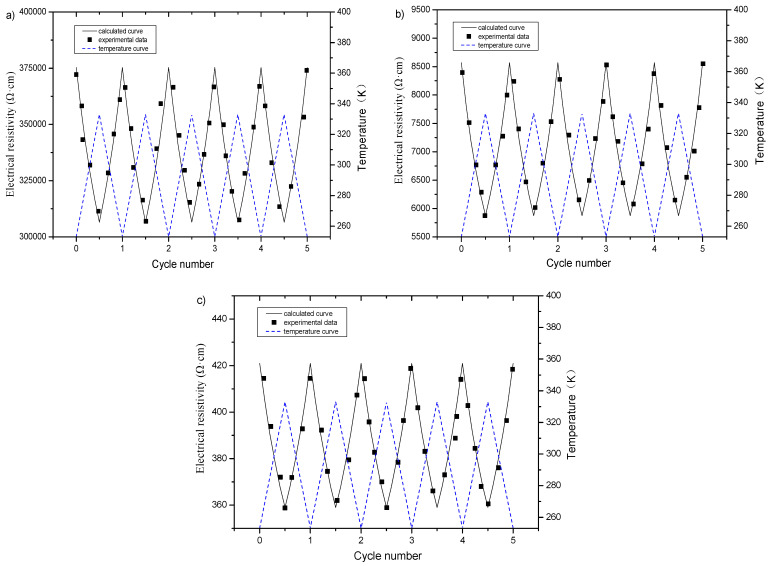
Calculated and measured value of electrical resistivity of specimens with different graphene contents subjected to multiple temperature rise and fall after freeze–thaw: (**a**) 1%; (**b**) 2%; (**c**) 3%.

The electrical resistivity of graphene-based cement composites after freeze–thaw has good regularity with temperature in the process of monotonic heating. In order to better reflect the stability of the law between electrical resistivity and temperature, the cement-based composites with graphene content of 1, 2 and 3% were subjected to multiple temperature rise and fall between 253 and 333 K after different freeze–thaw cycles. The resistivity values were measured and recorded at different temperatures and compared with the relationship between the resistivity and temperature fitted in the Section 3.3.2. The results are plotted in Figure 16. As observed, between 253 and 333 K, the measured value of electrical resistivity in each temperature rise and fall cycle is close to the calculated value, and its thermosensitive property possesses good stability, reversibility and repeatability.

Table 10 lists the temperature sensitivity of electrical resistivity of specimens before and after freeze–thaw at 273 to 333 K. This table indicates that the content of graphene and freeze–thaw action have great influence on temperature sensitivity of electrical resistivity, which decreases after freeze–thaw. Furthermore, the temperature sensitivity of resistivity of the specimen incorporating 2% of graphene is the highest, whether before or after freeze–thaw.

### 3.4. Pressure-Sensitive Property

#### 3.4.1. Influence of Freeze–Thaw on Electrical Resistivity under Monotonic Loading

**Figure 17:**
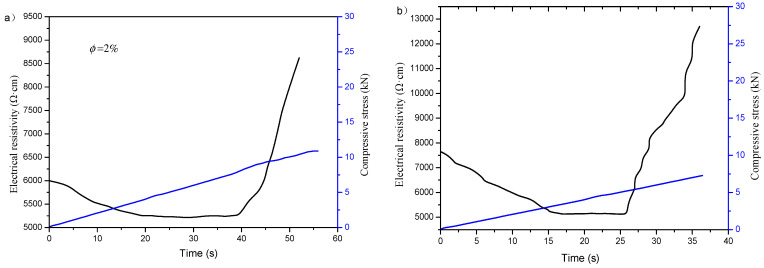
Relationship of electrical resistivity and compressive stress of specimens incorporating 2% content of graphene under monotonic loading: (**a**) before freeze–thaw; (**b**) after freeze–thaw.

Figure 17 shows the relationship between electrical resistivity and compressive stress of the specimen incorporating 2% content of graphene under monotonic loading after 50 freeze–thaw cycles. As can be seen from this figure, the response of electrical resistivity and pressure after freeze–thaw is similar to that before freeze–thaw. Specifically, the resistivity curve of the specimen after freeze–thaw under monotonic loading still contains three stages as before freeze–thaw: uniform decline period, stable platform period and rapid rise period. 

Here, the pressure sensitivity of graphene-based cement composites is defined as the quotient of the change rate of electrical resistivity under a certain stress and the resistivity of the composites without load, and the expression is:

(4) $$s=\frac{d\rho }{\rho_{0}dF}\times 100\%,$$

where *s* is the sensitivity (%/kN); *ρ*_0_ is the resistivity of the composites without load.

Due to uniform loading in this study, the relationship between load and time is linear, and the sensitivity can also be written as:

(5) $$s=\frac{d\rho }{\rho_{0}kdt}\times 100\%,$$

The pressure sensitivity of the uniform decline period before freeze–thaw is −3.5%/kN according to Equation (5), and it is −10.2%/kN after freeze–thaw, which is nearly two times higher, and the duration of the platform stability period is shortened by about half.

The cement-based composite will undergo volume deformation or even failure under pressure, which will affect the internal porosity and produce microcracks. In addition, the contact between conductive phases will also change. These factors will eventually cause changes in resistivity.

In the uniform decline period, the cement-based composite material produces strain under pressure. When it is in the range of linear elastic deformation, the strain changes monotonically but unevenly. It can be seen from Figure 17 that the resistivity of the composite basically decreases monotonically with the change of load, and is approximately linearly related. In this period, the composite is in the state of pressure compaction. The compressive stress shortens the distance between graphene particles and enhances the tunneling conduction, which is conducive to improving the internal conductive path and reducing the resistivity. The pressure sensitivity after freeze–thaw is greater than that before freeze–thaw, which is probably due to more microcracks and larger porosity caused by freeze–thaw, and thus greater change in the compaction process.

During the stable platform period, the pressure exceeds the proportional limit, and with the increase of pressure, new microcracks occur in the composite. The germination of fresh microcracks leads to destruction and reconstruction of the conductive network, so that the resistivity does not increase significantly. The curve maintains horizontal in a small range or oscillates up and down on the horizontal line.

In the rapid rise period, the pressure continues to increase and approaches the failure load. The cracks propagate and increase obviously, expand the distance between graphene, and destroy the conductive path, making the resistivity rises rapidly. After freeze–thaw, the duration of the stable platform period is shorter than that before freeze–thaw, which may be due to the larger porosity of the specimen after freezing and thawing, and the cracks are more likely to expand and increase [28,48].

#### 3.4.2. Influence of Freeze–Thaw on Electrical Resistivity under Cyclic Loading

##### Different Loading Amplitudes

**Figure 18:**
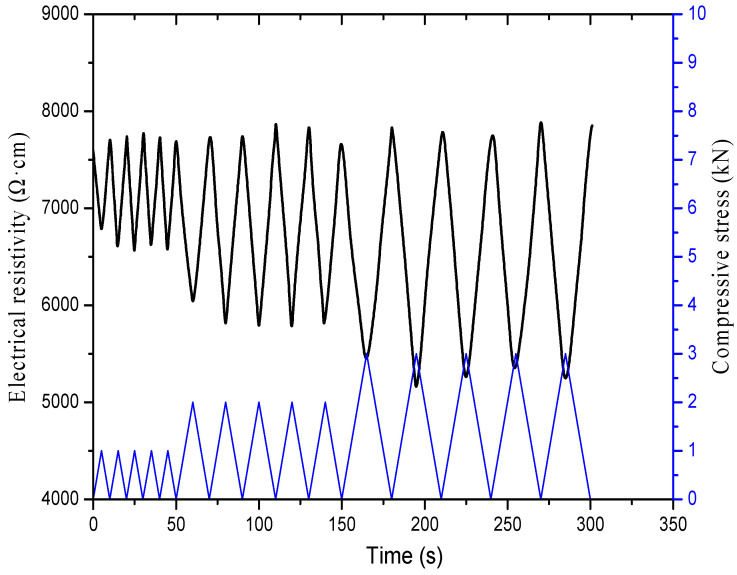
Relationship of electrical resistivity and compressive stress of specimens with 2% content of graphene under different loading amplitude after freeze–thaw.

Figure 18 displays the relationship between the electrical resistivity and the compressive stress of the specimen with 2% content of graphene after 50 freeze–thaw cycles. The loading and unloading rate is set as 0.2 kN/s, and the maximum loading pressures are 1, 2 and 3 kN, respectively. According to the monotonic loading results in the previous section, the cement-based composites incorporating 2% content of graphene are in a uniform decline period and within the elastic deformation range after freeze–thaw, when the maximum loading force does not exceed 3 kN. In the case of cyclic loading, under the maximum pressure load of each level, the minimum value of electrical resistivity at the first cycle is greater than the minimum value at multiple cycles, and the resistivity after unloading is greater than the initial value. Furthermore, the resistivity pressure curves of the specimens subjected to multiple cyclic loads are relatively consistent and have satisfactory repeatability under the same maximum pressure load of each level.

##### Different Loading Rates

**Figure 19:**
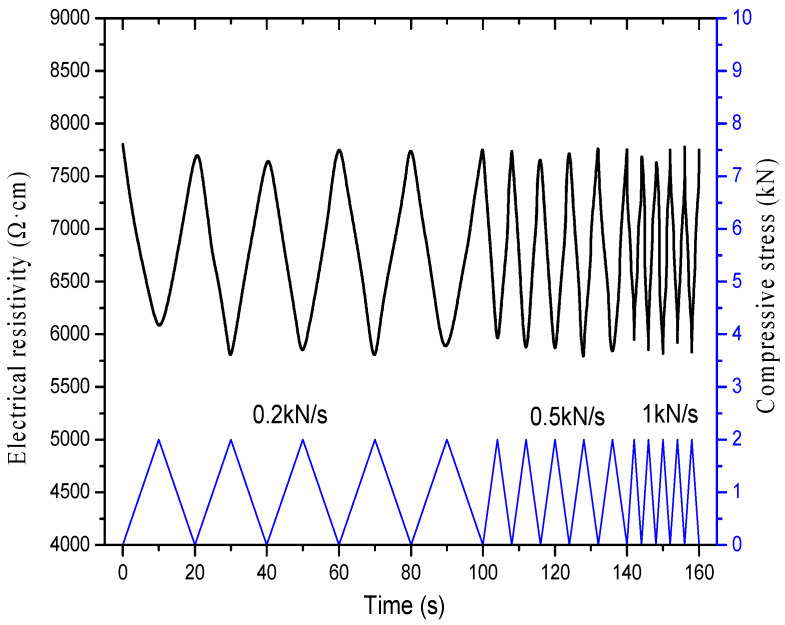
Relationship of electrical resistivity and compressive stress of specimens incorporating 2% content of graphene under different loading rate after freeze–thaw.

Figure 19 shows the relationship between electrical resistivity and compressive stress of specimens incorporating 2% content of graphene at loading rates of 0.2, 0.5 and 1 kN/s, respectively, after 50 freeze–thaw cycles. As presented in Figure 19, there is an excellent correspondence between the resistivity curve and the pressure curve at different loading rates, and the repeatability is favorable.

## 4. Conclusions

The freeze–thaw degree of graphene-based cement composites specimens after freeze–thaw treatment was characterized by freeze–thaw times. The influence of freeze–thaw on the electrical conductivity of graphene-based cement composites was investigated from two aspects of polarization effect and percolation threshold, and the mechanism of freeze–thaw effect on its electrical conductivity was analyzed. The effects of different humidity and temperature on the humidity sensitivity and thermosensitivity of the composites after freeze–thaw action were also studied. In addition, the effects of monotonic loading and cyclic loading on the pressure-sensitive property of composites after freeze–thaw were analyzed. Through the above research, the main conclusions are as follows:

(1). The influence of freeze–thaw on the electrical conductivity of graphene is mainly manifested in the influence on the electrical resistivity and the extension of the polarization time, and the influence on the percolation transition zone is small. Freeze–thaw leads to an increase in electrical resistivity, which is positively correlated with the number of freeze–thaw cycles.
(2). With the increase of freeze–thaw cycles, the cracks in the matrix increase, the crack width becomes larger, and the gel becomes loose. The water in the pores freezes and expands, making the pore size larger, generating new pores, and increasing the matrix porosity. More pores are generated in the matrix, which reduces the ionic conductivity of the composite with graphene content in zone A, increases the spacing of graphene particles in the composite with graphene content in zone B, resulting in a decrease in its tunneling conductivity, destroys the contact continuity of graphene particles in the composite with graphene content in zone C, and increases the resistivity of the specimens.
(3). After freeze–thaw, the polarization time of the specimen decreases with the increase of the relative water content, and the freeze–thaw action does not change the influence trend of relative water content on electrical resistivity. The resistivities of specimens incorporating graphene content of zone A and zone B decrease with the increase of water content after freeze–thaw, while the resistivity of specimens containing graphene content of zone C increases slightly with the increase of water content. For the specimens with graphene content of zone A or zone B and relative water content of 50 and 100%, respectively, ionic conduction in pore solution is an important form of their conduction. The microcracks caused by freeze–thaw increase the porosity, enhance the conductive network of pore solution, and reduce the resistance of specimens. The water content has little effect on the contacting conduction of the zone C specimen. The relationship between resistivity and water content of graphene-based cement composites under freeze–thaw action can be expressed by the improved Logistic function.
(4). The temperature has a great influence on the polarization effect of graphene-based cement composites after freeze–thaw, and the polarization time gradually shortens with the increase of temperature. In general, the electrical resistivity of the specimen decreases with the increase of temperature. Nevertheless, there is a temperature inflection point for specimen incorporating graphene content of zone B or zone C, and the resistivity increases with the increase of temperature after the inflection point. The reason is that before the inflection point, the thermal motion is dominant, which makes the resistivity decrease. After the inflection point, the change in material volume caused by temperature rise is the main factor, resulting in an increase in resistivity. The relationship between resistivity and temperature of graphene-based cement composites under freeze–thaw action can be expressed by the exponential function. In the temperature range of 273 to 333 K, the response of resistivity to temperature of composites after freeze–thaw is stable, reversible and repeatable, with good thermosensitivity and decreased sensitivity.
(5). After freeze–thaw, the composites incorporating graphene content of the zone B still show satisfactory pressure-sensitive property, and the sensitivity increases significantly with the degree of freeze–thaw. The compressive stress shortens the distance between the graphene particles in the specimens with graphene content of zone B, enhances the tunneling conduction, and leads to a decrease in resistivity. Freeze–thaw causes more microcracks and larger porosity of the composites, and the change in the compaction process is greater than that before freeze–thaw.

## Figures and Tables

**Table 1:** Composition of cement (wt.%).

Composition	CaO	SiO_2_	Al_2_O_3_	Fe_2_O_3_	MgO	SO_3_	Na_2_O	K_2_O	TiO_2_	MnO_2_	P_2_O_5_	LOI
Content	63.55	21.5	5.18	4.32	0.91	1.09	0.22	0.52	0.15	0.12	0.04	2.4

**Table 2:** Physical properties of cement.

Density (g/m^3^)	Specific Surface Area(m^2^/kg)	Standard Consistency Water Consumption(%)	Setting Time (min)		Compressive Strength(MPa)		Flexural Strength(MPa)	
			Initial	Final	3 Days	28 Days	3 Days	28 Days
3.18	360	25.4	136	222	26.4	52.1	5.2	8.3

**Table 3:** Composition of silica fume (wt.%).

Composition	SiO_2_	Al_2_O_3_	Fe_2_O_3_	MgO	Na_2_O	CaO
Content	85~95	1.1 ± 0.1	0.9 ± 0.2	0.7 ± 0.1	1.3 ± 0.2	0.3 ± 0.1

**Table 4:** Performance of graphene.

Purity (%)	Layers	Ratio of Monolayer (%)	Diameter (μm)	Diameter to Thickness Ratio	Bulk Density(g/mL)	Specific Surface Area(m^2^/g)
>98	1~3	>80	7~12	average 9500	0.01~0.02	50~200

**Table 5:** Mix proportions of graphene-based cement composites.

Specimen No	Cement (g)	Silica Fume (g)	Graphene (g)	Water (g)	Dispersant (mg)	Superplasticizer (g)	Defoamer (g)
G0	90	10	0	50	0	0	0
G0.5	90	10	0.5	50	15	0	0
G1	90	10	1	50	30	0	0
G1.3	90	10	1.3	50	39	0	0
G1.5	90	10	1.5	50	45	0	0.1
G1.7	90	10	1.7	50	51	0.2	0.1
G2	90	10	2	50	60	0.2	0.1
G2.3	90	10	2.3	50	69	0.2	0.2
G2.5	90	10	2.5	50	75	0.5	0.2
G2.7	90	10	2.7	50	81	0.8	0.3
G3	90	10	3	50	90	1	0.3

**Table 6:** Polarization time of specimens with different graphene content after different freeze–thaw cycles.

Graphene Content(%)	Number of Freeze–thaw Cycles	Polarization Time(min)
1.0	0	35
	25	35
	50	40
	75	45
	100	50
2.0	0	30
	25	35
	50	40
	75	40
	100	45
3.0	0	10
	25	10
	50	15
	75	20
	100	30

**Table 7:** Polarization time of specimens incorporating different graphene contents after freeze–thaw cycles at different relative water contents.

Graphene Content (%)	Relative Water Content (%)	Polarization Time (min)
1	0	40
	50	20
	100	12
2	0	40
	50	14
	100	10
3	0	15
	50	6
	100	4

**Table 8:** Polarization time of specimens with different graphene contents after freeze–thaw at different temperature.

Graphene Content (%)	Temperature (K)	Polarization Time (min)
1	273	45
	293	35
	313	30
	333	25
2	273	45
	293	40
	313	30
	333	25
3	273	20
	293	15
	313	15
	333	10

**Table 9:** Fitted results of electrical resistivity of specimens incorporating different graphene contents before and after freeze–thaw.

Graphene Content(%)	Before Freeze–Thaw			After Freeze–Thaw		
	*ρ_m_*	*b*	R^2^	*ρ_m_*	*b*	R^2^
1	147,093	247	0.9781	153,545	244	0.9674
2	889	558	0.9888	1619	455	0.9832
3	113	290	0.9958	207	194	0.9964

**Table 10:** Temperature sensitivity of electrical resistivity of specimens incorporating different graphene contents subjected to multiple temperature rise and fall before and after freeze–thaw.

Graphene Content(%)	Before Freeze–Thaw(%)	After Freeze–Thaw(%)
1	16.6	15.2
2	32.6	27.2
3	19.8	13.4

## Data Availability

The data presented in this study are available on request from the corresponding author.
